# Prep1, A Homeodomain Transcription Factor Involved in Glucose and Lipid Metabolism

**DOI:** 10.3389/fendo.2018.00346

**Published:** 2018-06-28

**Authors:** Francesco Oriente, Giuseppe Perruolo, Ilaria Cimmino, Serena Cabaro, Antonietta Liotti, Michele Longo, Claudia Miele, Pietro Formisano, Francesco Beguinot

**Affiliations:** ^1^Department of Translational Medicine, Federico II University of Naples, Naples, Italy; ^2^URT Genomic of Diabetes, Institute of Experimental Endocrinology and Oncology, National Research Council, Naples, Italy

**Keywords:** transcription factors, Prep1, TALE proteins, insulin signaling, diabetes mellitus

## Abstract

The three-amino acid loop extension (TALE) homeodomain proteins are a family of transcription factor including the mammalian Pbx, MEIS and Prep proteins. TALE proteins can bind other transcription factors such as Pdx-1 and play an important role in the regulation of glucose metabolism. Experiments performed in mutant mice have shown that while the single *Pbx1* or *Pdx-1* knockout mice feature pancreatic islet malformations, impaired glucose tolerance and hypoinsulinemia, the trans-heterozygous *Pbx1*^+/−^ *Pdx1*^+/−^ mice develop age-dependent overt diabetes mellitus. In contrast, Prep1 plays a different role with respect to these proteins. Indeed, *Prep1* hypomorphic mice, expressing low levels of protein, feature pancreatic islet hypoplasia accompanied by hypoinsulinemia similar to Pbx1 or Pdx1. Nevertheless, these animals show increased insulin sensitivity in skeletal muscle, liver and adipose tissue accompanied by protection from streptozotocin-induced diabetes. In addition, *Prep1* hypomorphic mice feature reduced triglyceride synthesis and do not develop steatohepatitis after a methionine and coline deficient diet. In this review we have underlined how important metabolic functions are controlled by TALE proteins, in particular by Prep1, leading to hypothesis that its suppression might represent beneficial effect in the care of metabolic diseases.

## Introduction

Diabetes mellitus (DM) is a metabolic disease due to a combination of genetic and environmental factors. Previous studies performed by candidate gene approach, family linkage studies, and gene expression profiling discovered a number of DM genes, but the genetic basis of common diabetes still lack complete explanation (1). Several genes potentially involved in the onset of DM encode for transcription factors (TF). Among them, mutations in hepatocyte nuclear factors HNF4α, HNF1α, HNF1β, insulin promoter factor IPF1, and NeuroD genes may induce different hereditary forms of diabetes mellitus such as the maturity onset diabetes of the young (MODY) (2). Furthermore, genetic variants of another transcription factor, the peroxisome proliferative-activated receptor γ (PPARγ), have been associated to insulin-resistance and type 2 diabetes, the most common form of DM (3–5).

In the present review, we will focus on the metabolic role of TALE (Three Aminoacid Loop Extension) transcription factors and, in particular, on a gene belonging to this family named *Prep1*.

### TALE proteins

**Figure 1:**
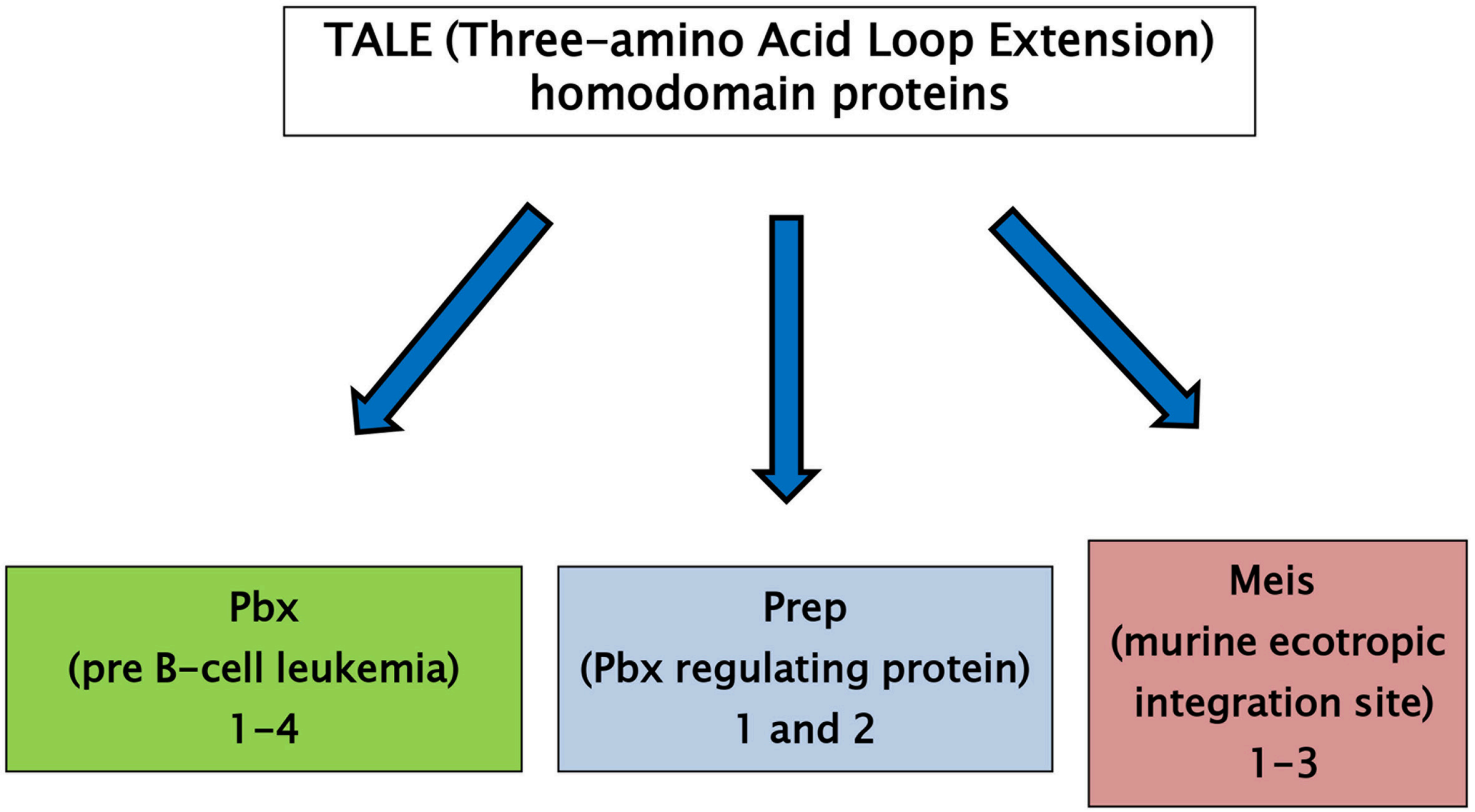
TALE (Three-amino Acid Loop Extension) homeodomain proteins are divided into two groups: the PBC family, including the vertebrate Pbx proteins, fly Extradenticle and worm Ceh-20, and the MEIS family, including vertebrate Meis and Prep proteins, fly Homothorax (Hth) and worm Unc-62.

The three-amino acid loop extension (TALE) homeodomain proteins have been described as transcription factors involved in regulation of growth and differentiation occurring on embryo development. TALE genes are highly conserved in the common ancestor of plants, fungi, and animals. TALE proteins display a highly conserved DNA binding domain of approximately 60 amino acids called the homeodomain (6). This region is composed of three alpha helices and a flexible N-terminal arm. The homeodomain interacts with the DNA through the third helix making base-specific contacts in the major groove of DNA and through the N-terminal arm which contacts the minor groove of DNA. Between the first and the second alpha helices of the homeodomain there is an extension of three amino acids, represented by a proline (P)—tyrosine (Y)—proline (P) in position 24–26. This domain is involved in important protein–protein interactions playing a major role in development (7, 8). TALE homeodomain proteins can be classified in two groups: the PBC family, which includes the vertebrate Pbx proteins, fly Extradenticle, and worm Ceh-20, and the MEIS-MEINOX family, including vertebrate Meis and Prep, fly Homothorax (Hth) and worm Unc-62 (Figure 1) (8, 9). A comprehensive description of ontogenic relations between the PBC/Meis-Meinox family members has been recently reported (9).

### Pbx proteins

**Figure 2:**
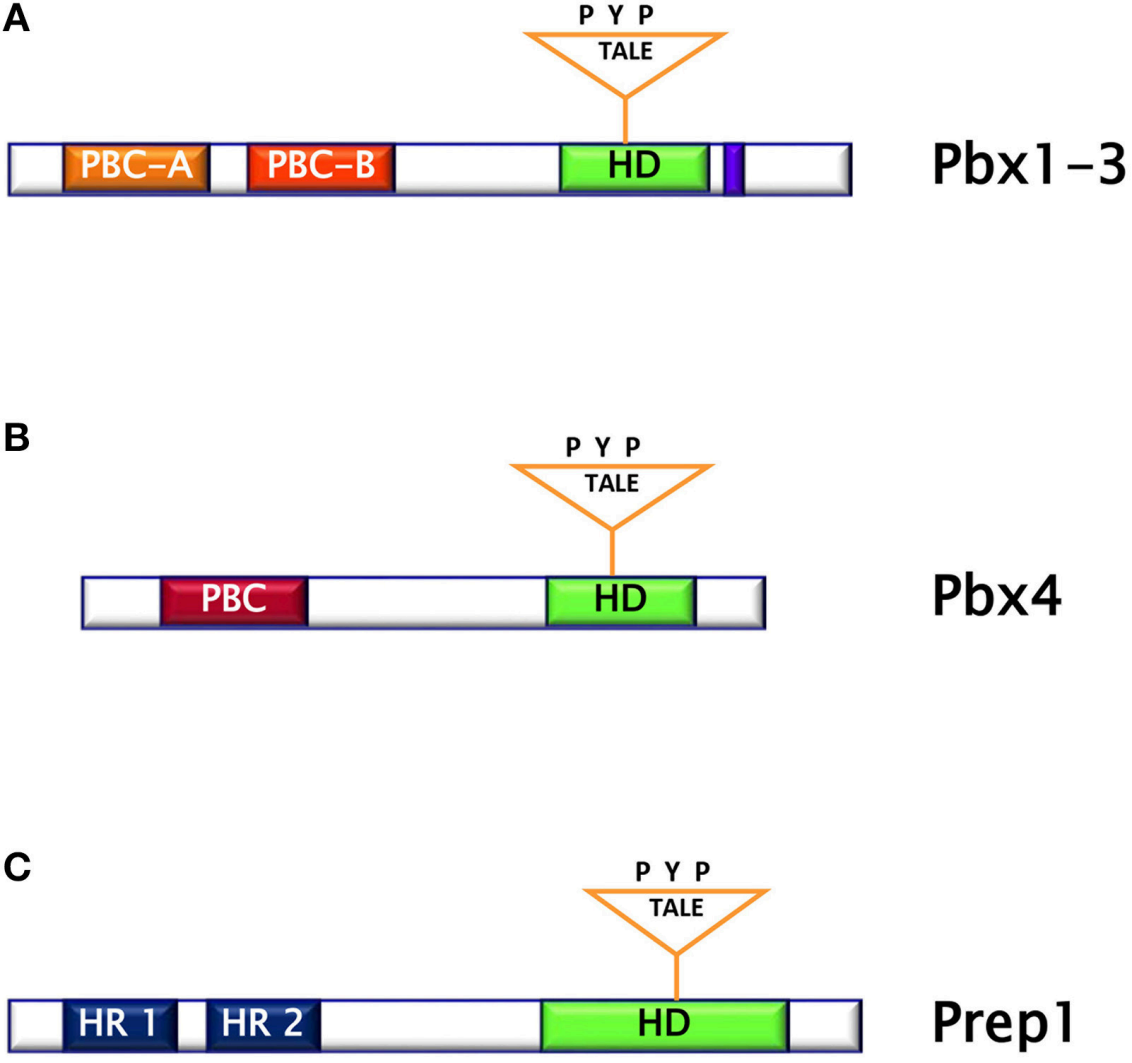
**(A)** Pbx1-3 protein is characterized by the homeodomain region including the three aminoacid loop extension and by two highly homologous regions named PBC (Pbx and Ceh-20) **(A,B)**. **(B)** Pbx4 protein is smaller than Pbx1-3 and bears an homeodomain at the C-terminus. **(C)** Prep1 protein is characterized by the homeodomain region including the three aminoacid loop extension and by two sequences similar to MEIS named HR (Homology Region) 1 and 2.

The PBC subclass comprises the proteins Pbx1 (Pbx1a and b), Pbx2, Pbx3 (Pbx3a-d), and Pbx4. Pbx are characterized by the DNA-binding homeodomain region including the three aminoacid loop extension and by two highly homologous regions named PBC- A and -B important for protein-protein interaction. Pbx share extensive sequence identity both within and flanking their homeodomains (up to 97% within their homeodomains) (10). However, Pbx1-3 contain 78 additional residues, which include part of the PBC-A domain, and a 30-residues stretch in the C-terminal domain, as compared to Pbx4. (Figures 2A,B). The differences between Pbx proteins are also evident in their expression pattern. Indeed, while Pbx1a isoform is restricted to the brain, Pbx1b is expressed in the whole body. Pbx2 protein is detected in nuclear and cytoplasmic fractions of endocrine and acinar cells, while Pbx3b is present only in the cytoplasmic fractions of both cell types (11). In contrast, Pbx4 is expressed exclusively in the testis (12). Several experiments performed in mice have indicated that the individual contribution of each member of PBC family seems to be different because, indeed, while *Pbx1* single mutant has a wide range of malformations, only minor or no phenotypes are obvious in *Pbx2* and *Pbx3* mutants, suggesting a major role for Pbx1 in mammals (13). In particular, these studies have evidenced the role of Pbx1 in the development and metabolism. Indeed, Pbx1-deficient mice die at E15.5, displaying several defects including hypoplasia, aplasia or ectopia of multiple organs, as well as widespread defects of the axial and appendicular skeleton (13). Skeletal malformations are observed in the proximal elements of limbs, in ribs and vertebrae and the skeletal structures of the second branchial arch undergo an anterior homeotic transformation into first arch-derived cartilages. Examination of *Pbx1*^+/−^ mice has also shown that this transcription factor is required for pancreatic insulin secretion in mature islets, as *Pbx1* inactivation causes reduction of circulating insulin levels and impaired glucose tolerance, conditions known to presage the onset of overt type 2 diabetes. In these animals, the levels of PDX-1 are strongly reduced, indicating that Pbx1 is important for its expression and most probably assessing the molecular events responsible for the observed phenotype (13–15). Studies performed in transheterozygous *Pbx1*^+/−^*/Pdx1*^+/−^ mice have indicated that genetic interactions between Pbx1 and Pdx1 regulate postnatal islet structure and function. Consequently, simultaneous haploinsufficiency for both *Pbx1* and *Pdx1* results in an oligogenic mouse model of age-dependent diabetes mellitus (15). Among the PBC cofactors, the TALE homeoprotein Prep1 is important for the target specificity and regulatory function of Pbx1 (8).

### Prep proteins

**Figure 3:**
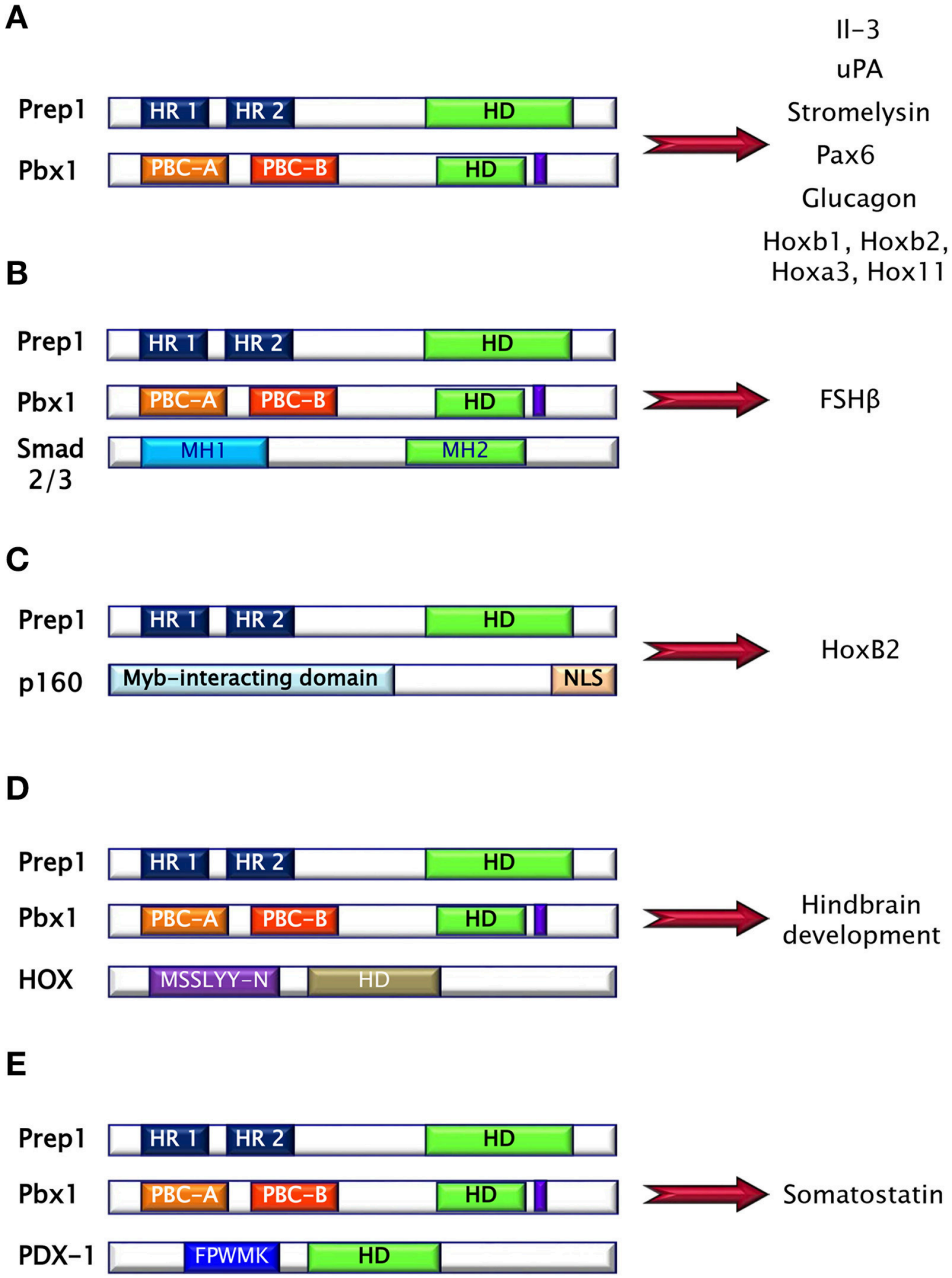
Prep1 protein may form binary and ternary complexes with several proteins, mainly via HR domains. **(A)** Pbx1-Prep1 complex controls the gene expression of the interleukin 3 (IL-3), stromelysin, urokinase plasminogen activator (uPA), Pax6, glucagon and several HOXs. **(B)** Pbx1-Prep1 complex, in collaboration with the transcription factors Smad2 and 3, induces the actvin-mediated transcription of the beta subunit of follicle stimulating hormone (FSH?). **(C)** Prep1-p160 complex inhibits Prep1-dependent HoxB2 expression. **(D)** Prep1/Pbx/HOX heterotrimers is required for hindbrain development. **(E)** Prep1/Pbx/PDX-1 is necessary for somatostatin gene transcription.

Prep (Pbx regulating protein) is a collective name for homeodomain transcription factors belonging to the MEINOX subfamily of the TALE proteins expressed in several tissues (16). Vertebrates express two genes, *Prep1* and *Prep2*, while three genes, named *Prep1.1, Prep1.2*, and *Prep2*, have been found in zebrafish. In mouse, *Prep1* gene maps on chromosome 17 and encode for a 64 kDa protein which can localize both in cytoplasm and in nucleus (16). The homeodomain region necessary for DNA binding has been localized near the C-terminus and between the first and the second alpha helices of the homeodomain there is the 3 aminoacid loop extension (TALE) (Figure 2C) (16, 17). Prep1 can form heterodimers with PBC proteins and, in particular, sequences of Prep similar to other MEIS proteins named HR (homology region) 1 and 2 bind the PBC-A sequence of Pbx. Heterodimerization with Pbx appears to be essential to translocate Prep into the nucleus, as neither Prep1 and Prep2 contains a nuclear localization signal. On the other hand, Prep1 dimerization prevents nuclear export and the proteasomal degradation of Pbx prolonging its half-life (17–19). Heterodimerization of Prep1 with Pbx1 forms the UEF-3 (urokinase Enhancer factor-3) transcription factor which controls the expression of the interleukin 3 (IL-3), stromelysin, and urokinase plasminogen activator (uPA), a protease involved in fibrinolysis, innate and adaptive immunity (16). In addition, coexpression of Pbx1-Prep1 inhibits the glucagon promoter in non-glucagon-producing cells and regulates several HOX expression (Figure 3A) (20), while in collaboration with the transcription factors Smad2 and 3, Pbx1-Prep1 complex induces the actvin-mediated transcription of the beta subunit of follicle stimulating hormone (FSHβ) (Figure 3B) (21). Blasi and coworkers have identified another direct Prep1-interacting protein, p160 Myb-binding protein (p160), that competes with Pbx1 for Prep1 binding and inhibits Prep1-dependent HoxB2 expression (Figure 3C) (22). Thus, Prep1 functions may depend not only on its interaction with Pbx, but also with p160. Prep1 and Pbx1 can form ternary complexes with HOX proteins or the pancreatic and duodenal homeobox 1 (PDX1). The role of Prep1/Pbx/HOX heterotrimers is required for Hox-dependent gene regulation, particularly in the development of hindbrain (Figure 3D) (23, 24), while PDX-1, also known as insulin promoter factor 1 (IPF1), is necessary for somatostatin gene transcription (25) (Figure 3E).

While Prep1 has been well characterized, not many information are available about Prep2. This gene maps on chromosome 11 and encodes for a 70 kDa protein expressed in cytoplasm and in the nucleus. Prep2 is larger than Prep1 (461 vs. 436 residues) and displays a 52% homology with Prep1, but lacks similarity to any MEIS family members (17).

Different “*in vivo”* observations have underlined a key role of Prep1 in organs and tissues development. In zebrafish, reduction of *prep1.1* gene expression causes embryonic lethality due to extensive brain apoptosis, loss of hindbrain rhombomeric segmentation, lack of cartilage differentiation of neural crest cells, pericardial edema, and lack of fins (26). Similarly, in mice, a null *Prep1* mutation results in early mortality (E7.5) (27), precluding a study of the *Prep1* roles in later developmental processes. To overcome this problem, *Prep1* hypomorphic (*Prep1*^*i*/*i*^) and *Prep1* heterozygous (*Prep1*^*i*/+^) mouse models, expressing 3–7 and 55–57% of the protein, respectively, have been generated. Most *Prep1*^*i*/*i*^ embryos die between E17.5 and P0, although about 1/4 of these escape embryonic lethality (28). Mice escaping embryonic lethality show T-cell development anomalies (29). In addition, erythropoiesis and angiogenesis are impaired, with liver hypoplasia, decreased hematocrit, anemia, and delayed erythroid differentiation together with a decrease in capillary formation. *Prep1*^*i*/*i*^ embryos also display major eye anomalies and exhibit decreased levels of Pbx1, Pbx2, and Meis1 proteins as well as decreased expression of *cMyb* and *Pax6*, consistent with the hematopoietic and eye phenotype, respectively (28).

A wide body of literature has underlined the irreplaceable involvement of Prep1 in brain development. In human, *Prep1* maps at chromosome 21q.22.3 and is expressed in triple copy in Down's syndrome (DS) patients. Overexpression of Prep1 causes fatty acid binding protein (FABP)-7 promoter trans-activation in cultured neuroblastoma cells and as, FABP7 function is necessary for neuro- and glio-genesis, particularly for the establishment of the glial fibers and the proper migration of immature neurons to cortical layers, it is possible that Prep1-mediated *FABP7* overexpression may contribute to the DS-associated neurological disorders (30). *In situ* hybridization analysis on zebrafish embryos has shown that *prep1* gene is ubiquitously expressed up to 24 h post-fertilization (HPF) and restricted to the head from 48 HPF onwards where it is critically involved in apoptosis and differentiation processes during neuronal crest cell differentiation and in craniofacial chondrogenesis (26). It has been demonstrated that, during zebrafish embryogenesis, the formation of Prep1-Pbx1-Hoxb1/2 trimeric complex, through the Pbx-Meis binding (PM) site, represents an essential support for Hoxb(s) in segmentation of r3 and r4 rhombomeres (31). Studies performed on mouse embryos have also confirmed that Prep1 is deeply expressed in hindbrain since day 9.5 and that it confers high stability to Prep1-Pbx1-Hoxb1 multimeric complex in r4 rhombomere development (23, 28, 32). Our recent data (33) have evidenced that *Prep1* deficiency alters olfactory system morpho-functional integrity. Indeed, brain morphological analysis has revealed that *Prep1*^*i*/+^ mice show a significant reduction of olfactory bulb (OB) area, a reduced number of periglomerular interneurons and an increased number of mitral cells within the main olfactory bulb, compared to WT mice. In addition, *Prep1*^*i*/+^ mice feature a reduced neuronal metabolism and a low ability to distinguish odor scents. Molecular analysis, indicate that *Prep1*^*i*/+^ mice displays significantly reduced BDNF signaling and suggests that Prep1 promotes neuronal cell viability and function by controlling TrkB-mediated pathway.

## Prep1 and metabolism

*Prep1* hypomorphic mice have a complex metabolic phenotype. Indeed, these animals show smaller pancreatic islet but normal architecture compared to their littermates. In addition, *Prep1*^*i*/*i*^ mice feature islet hypoplasia and absolute reduction of insulin secretion. The *Prep1*^*i*/*i*^ phenotype also includes a strong reduction of pancreatic *Pbx1* expression, emphasizing the concept that Prep1 hierarchically acts upstream in the network regulating pancreas development by controlling the levels of Pbx1. However, these mice are protected from streptozotocin-induced diabetes and display enhanced peripheral insulin sensitivity, as indicated by measurement glucose uptake and insulin-dependent glucose disposal (34).

### Skeletal muscle

*Prep1*^*i*/*i*^ muscle phenotype does not depend on reduced expression of *Pbx1*, but on reduced levels of the p160 Myb-binding protein (p160), a molecule which is known to inhibit the PGC-1α signaling. Low levels of Prep1 in the muscle of hypomorphic mice determines a significant decrease of p160, accompanied by enhanced expression of the PGC-1α and GLUT4 transporter. This, in turn, leads to increased insulin-stimulated glucose uptake. These effects have been analyzed more in detail by transfecting *Prep1* in L6 skeletal muscle cells. Overexpression of Prep1 stabilizes p160, inducing p160 escape from proteasome, and reduces the levels of PGC-1α and GLUT4, impairing glucose transport (34). Other two intriguing studies have suggested that both an abnormal level of glucose or lipids, often accompanying insulin-resistance, may upregulate Prep1 levels by different mechanisms. Ciccarelli et al. indicate that high glucose exposure of L6 skeletal muscle cells can induce NF-κB activation and histone modification at the Prep1 5′ region, leading to enhanced transcription of Prep1. Thus, following Prep1 upregulation, the repressor complex myocyte enhancer factor 2 (MEF2)/histone deacetylase 5 (HDAC5) is recruited at the GLUT4 promoter and reduces GLUT4 expression (35). More recently, Cimmino et al have shown that treatment of L6 cells with ceramides, a family of lipids inducing insulin-resistance, increases the levels of Prep1 and p160 and promotes their association. Furthermore, these lipids as well as Prep1 overexpression, strongly reduce insulin-mediated IR, IRS1 and Akt phosphorylation and PGC-1α and GLUT4 protein expression, leading to the inhibition of glycogen synthesis and glucose uptake. Interestingly, prevention of Prep1-p160 binding by a synthetic Prep1(54–72) peptide, mimicking the Prep1 region involved in the interaction with p160, reduces this association and increases PGC-1α and GLUT4 levels.

In addition a Prep1(54–72)-mediated Prep1-p160 complex disruption restores IR-IRS1 tyrosine phosphorylation impaired by ceramide treatment or Prep1 overexpression. These data indicate that, in L6 cells, Prep1 impairs metabolic effects through two distinct mechanisms, one involving the downregulation of PGC-1α and GLUT4, the other, involving direct impairment of insulin pathway (36).

### Liver

The mechanisms responsible for Prep1 action in liver differ from those in the skeletal muscle since Pbx1 and p160 are expressed at different levels. In fact, while there is a marked reduction of p160 in the skeletal muscle of the hypomorphic mice compared to the wild type animals, this difference has not been detected in liver mostly likely because the expression of p160 is very low in this organ. Thus, at variance with muscle, Prep1 major functional partner in liver appears to be Pbx1 rather than p160. Prep1 reduction in liver improves insulin signaling increasing hepatic glycogen content and decreasing glucose output and triglyceride levels. Analysis of insulin signal transduction in the *Prep1*^*i*/*i*^ mouse liver revealed increased tyrosine phosphorylation of both insulin receptor and the major IRSs. These effects are paralleled by a significant reduction of expression of SHP1 tyrosine phosphatase. Further experiments in HepG2 liver cells stably transfected with *Prep1* revealed a significantly decreased insulin effect on IR and IRSs tyrosine phosphorylation and on glycogen accumulation. In particular, expression of SHP1 in the cellular models negatively correlates with insulin signaling. However, antisense silencing of *SHP1* rescues insulin action, suggesting a functional relevance for *SHP1* as a Prep1 target in the liver. Experiments performed “*in vitro”* have suggested that the expression of both Prep1 and Pbx1 displays a powerful enhancer function on *SHP1* gene expression. Thus, Prep1-Pbx1 complex induces *SHP1* gene transcription and impairs insulin signaling (37). Kulebyakin et al. have further investigated the molecular mechanism by which Prep1 may cause hepatic insulin-resistance. The authors suggest that Prep1 stimulates hepatic glucose production by stabilizing the nuclear localization of Foxo1 and increasing the expression of two major gluconeogenic enzymes, phosphoenolpyruvate carboxykinase 1 and glucose-6-phosphatase (38). *Prep1* action in liver involves also lipid synthesis (39). Indeed, in parallel with the reduction of hepatic triglyceride content, also serum triglyceride levels are strongly reduced in *Prep1*^i/+^ mice. FAS expression, an enzyme that regulates *de novo* hepatic lipogenesis, is significantly reduced in *Prep1*^*i*/+^ mice. Consistent with these data, the molecular pathway controlling hepatic lipogenesis is downregulated. In fact, *Prep1*^i/+^ mice feature increased phosphorylation of PKCζ, LKB1, AMPK, and ACC, leading to an inhibition of TG synthesis. This regulation is due to the modulation of expression of SHIP2, a lipid phosphatase, known inhibitor of PI3Kinase/PKCζ signaling. In the liver of *Prep1*^*i*/+^ mice, SHIP2 protein and mRNA expression, is strongly reduced. Accordingly with these data, HepG2 cells overexpressing *Prep1* display increased triglyceride levels and FAS expression, PKCζ, LKB1, AMPK, and ACC phosphorylation is strongly reduced, while SHIP2 levels are increased. Interestingly, overexpression of Pbx1 cDNA in HepG2 cells mimics *Prep1*-induced triglyceride synthesis. At the opposite, *Prep1*_HR1_ mutant, which is unable to bind Pbx1, fails to elicit these effects. ChIP (and Re-ChIP) experiments indicate that Prep1/Pbx1 complex can bind SHIP2 promoter region and regulate its expression. Liver damage and intracellular TG content induced by treatment with a steatogenic diet is much less pronounced in *Prep1*^*i*/+^ mice compared to their wild type littermates, indicating that *Prep1* silencing protects mice from diet-induced steatohepatitis (39).

### Adipose tissue

Adipose tissue acts not only as the major fat storage site, but also as an endocrine organ and its alterations may contribute to different human disorders such as cardiovascular diseases, type 2 diabetes and obesity. We and others have also evidenced an important role of Prep1 in adipogenesis (40, 41). Adipocyte differentiation requires a time-regulated sequence of gene-expression events. It has been suggested that impaired adipogenesis may lead to developments larger fat cells, insulin resistance, and eventually type 2 diabetes (42). Our data indicate that *Prep1*^*i*/+^ mice feature higher expression of several adipogenic markers, as *C/EBP*α, *C/EBP*β, *FABP4, GLUT4*, and *PPAR*γ*2* compared to the WT animals. How Prep1 affects adipogenesis is not clear. Maroni et al. have recently reported that *Prep1* downregulation increases C/EBPβ binding to chromatin, predisposing the adipocytes toward adipogenic differentiation (40).

Our very recent data (41) indicate that *Prep1*^i/+^ mice feature significantly decreased visceral adipose mass and adipocyte cell area. In turn, an increased number of small size adipocyte has been detected in the white adipose tissue from *Prep1*^i/+^ mice, accompanied by a significant increase of insulin signaling. In addition, Prep1 deficiency decreases T cell infiltration and pro-inflammatory cytokines expression and secretion. At variance, the expression and secretion of adiponectin, a positive regulator of insulin signaling, is strongly increased in *Prep1*^i/+^ mice. Experiments performed in murine liver cells incubated with conditioned media obtained from mature adipocytes isolated from WT and *Prep1*^i/+^ mice indicate that adipocyte-released factor of *Prep1*^i/+^ mice improves glucose metabolism and insulin signaling, suggesting that the impact of Prep1 on hepatic glucose homeostasis is mediated not only by a direct action on the organ but also through a crosstalk between adipocytes and hepatic cells.

## Conclusions

**Figure 4:**
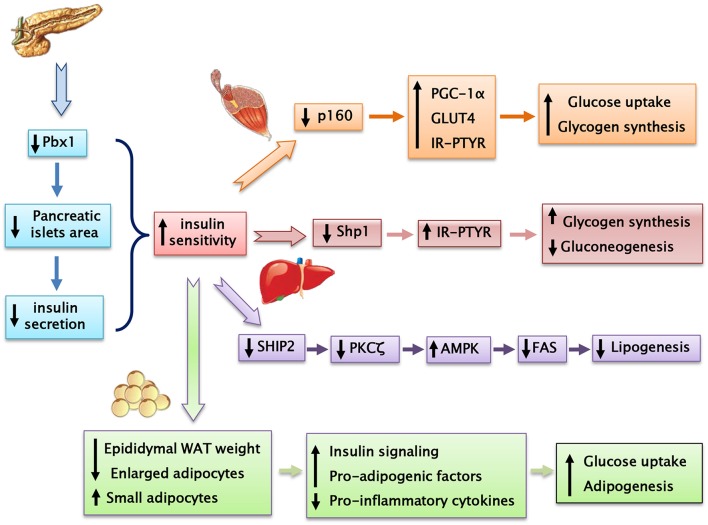
Prep1 hypomorphic mouse, despite having a reduced insulin secretion, shows a better peripheral insulin sensitivity compared with their wild-type littermates.

Type 2 diabetes (T2D) is the most common form of diabetes accounting for 90% of all diabetics. It is estimated that 55,000,000 adults in Europe have T2D, which is expected to reach 66,000,000 in 2030. For decades, antihyperglycemic agents have been used for the treatment of type 2 diabetes mellitus given their effectiveness and convenience. Although their efficacy and safety are quite well documented, these drugs are not totally free of undesired events. Thus, the discovery of new molecules is crucial to envision new targeted strategies for preventing or treating type 2 diabetes and its related diseases. We have identified *Prep1* as a gene encoding for a homeodomain transcription factor which induces muscular, hepatic and adipose tissue insulin-resistance (Table 1). The relevance of these findings has been highlighted by the results obtained in Prep1 hypomorphic mice expressing low levels of protein which are protected from streptozotocin-induced diabetes and show improved peripheral and hepatic glucose and lipid metabolism (Figure 4). Much remains to be uncovered about *Prep1* action, and, in particular, whether Prep1 could have a clinical relevance in the treatment of insulin-resistance and prevention of type 2 of diabetes in humans. However, the discovery and the characterization of Prep1 as a new regulator of metabolism opens to the possibility of being a target for improving and/or treating metabolic diseases.

**Table 1:** Metabolic role of Prep1.

**Organ**	**Principal interactor**	**Gene expression**	**Metabolic effects**	**References**
Skeletal muscle	p160	↓ Glut4	↓ Glucose uptake ↓ Glycogen synthesis	(34) (35) (36)
Liver	Pbx1	↓ Shp1 ↓ SHIP2	↓ Glycogen synthesis ↓ Lipogenesis	(37) (38) (39)
Adipose tissue	Pbx1	↓ C/EBPβ	↓ Glucose uptake ↓ Adipogenesis ↑ Inflammation	(40) (41)

*↑, increase; ↓, decrease*.

## Author contributions

FO and GP prepared the first draft of the manuscript. IC, SC, AL and ML were involved in the literature search. CM and PF critically revised the manuscript. PF and FB supervised the work and wrote the final version of the article.

### Conflict of interest statement

The authors declare that the research was conducted in the absence of any commercial or financial relationships that could be construed as a potential conflict of interest. The reviewer MH and handling Editor declared their shared affiliation.
